# Creeping Cold Nodules: Ascending Lower Extremity Nodules in an Immunocompromised Patient

**DOI:** 10.7759/cureus.69721

**Published:** 2024-09-19

**Authors:** William H Hewlett, Adam M Garber

**Affiliations:** 1 Internal Medicine, University of Pennsylvania, Philadelphia, USA; 2 Internal Medicine, Virginia Commonwealth University School of Medicine, Richmond, USA

**Keywords:** ascending pattern, cold nodules, mycobacterium chelonae, non-tuberculosis mycobacteria, renal transplant patient

## Abstract

Non-tuberculous mycobacterium (NTM) infection can cause a broad range of pathology, especially in an immunocompromised patient. We report a case of ascending lower extremity nodules in a renal transplant patient that were found to be caused by *Mycobacterium chelonae*, an NTM. We discuss the clinical evaluation and workup of this patient and why NTM infections may require a high degree of clinical suspicion to properly diagnose and treat.

## Introduction

Non-tuberculous mycobacterial (NTM) can infect a variety of tissues and are caused by over 170 organisms [1], 91 of which have been identified in human samples [2]. These organisms are extremely hardy and pervasive throughout our environment. They are often associated with infection in immunocompromised individuals; however, some studies suggest an increasing incidence in immunocompetent individuals [3]. Due to their atypical characteristics, including slow-growing nature and acid-fast bacilli (AFB) staining, these infections often remain undetected.

Over 90% of NTM infections cause pulmonary disease [4], with estimated rates of pulmonary NTM infection in North America ranging from 1 to 15 per 100,000 persons [5]. However, NTM can also cause extra-pulmonary infections, with annualized prevalence cited at 7.5 to 11 cases per 100,000 inpatients [6,7]. Skin and soft tissue infections have been noted to have the highest prevalence of extra-pulmonary NTM infections, followed by disseminated NTM infections (4.4 vs. 3.7 cases per 100,000 hospitalized patients, respectively) [6]. NTM infections can present many ways, with one recent case report describing a case of NTM tenosynovitis [8]. The mortality rate of NTM is estimated to be 2.3 deaths per 1,000,000 person-years [9].

This case describes NTM masquerading as ascending, subacute soft tissue nodules in an immunocompromised patient and highlights alternative differential diagnoses to consider.

## Case presentation

A 62-year-old man with end-stage renal disease secondary to diabetes mellitus, who underwent a deceased-donor kidney transplant two years prior to presentation on immunosuppressive therapy with prednisone and tacrolimus, presented to the hospital with progressively painful, ascending nodules on his right lower leg over the past three to four weeks. He was recently seen by his primary care physician for the nodules on his ankle and distal lower extremity and was given seven days of trimethoprim-sulfamethoxazole, which he completed approximately five days prior to presenting to the hospital. Despite the antibiotic, the nodules continued to spread up his medial right leg, one of which spontaneously drained purulent fluid. He denied any systemic symptoms, including fever or chills. He reported compliance with all his prescribed medications. He had no new exposures or injuries.

**Figure 1:**
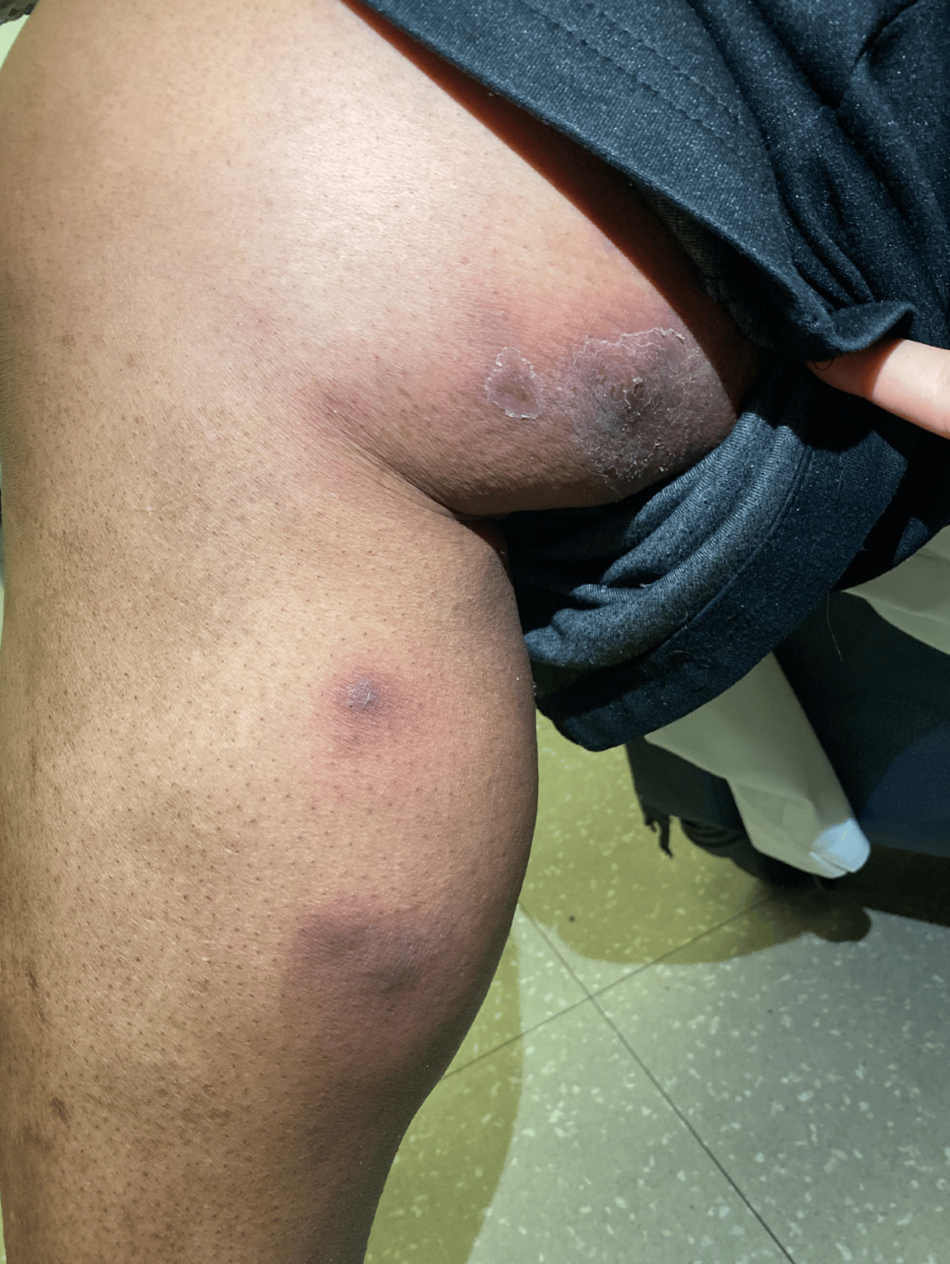
Image of the ascending, grouped cold nodules

**Figure 2:**
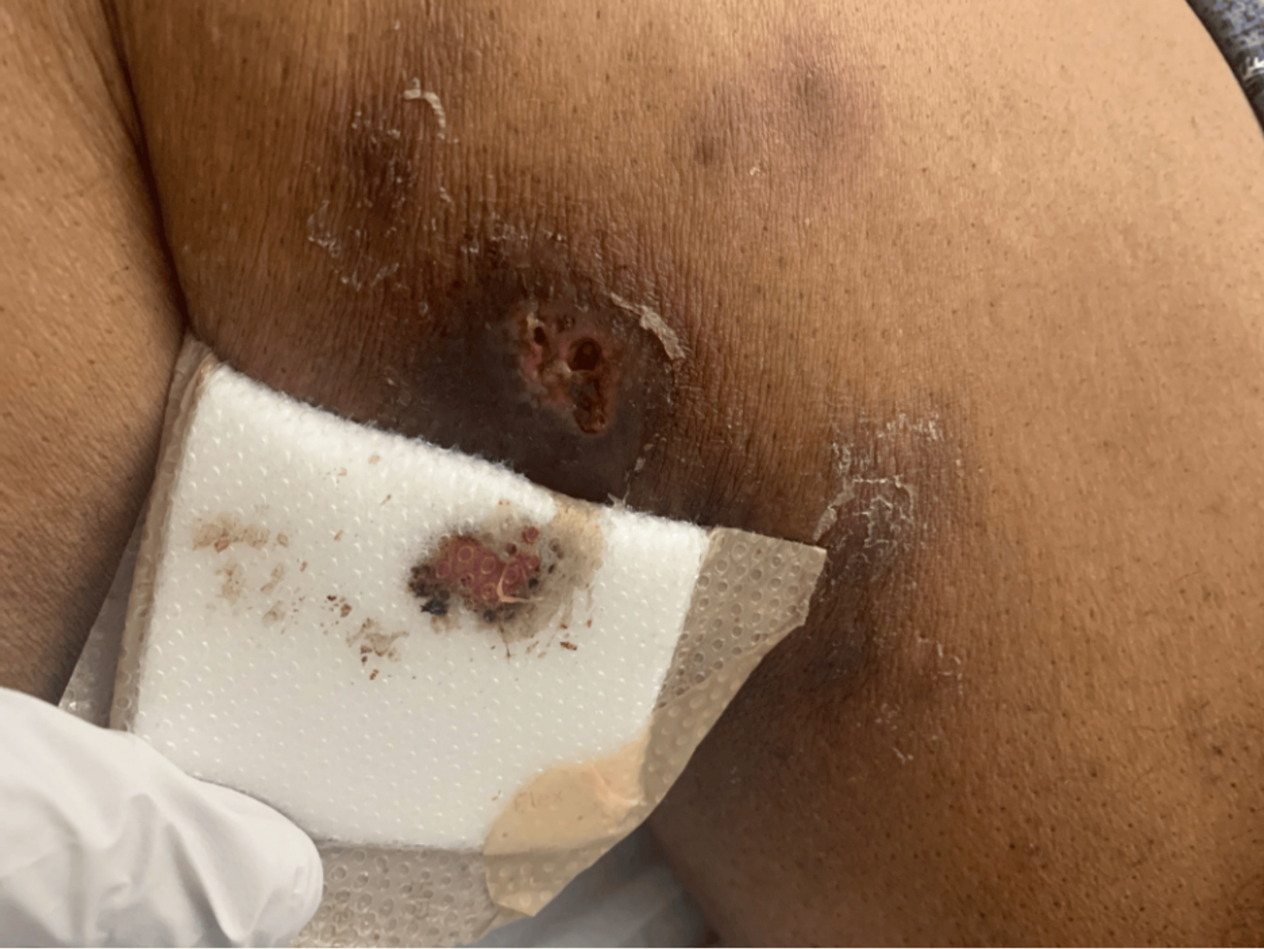
Ulcerative lower leg lesion

The initial exam was significant for nodules in groups of three or fewer that "skipped" along the medial aspect of the right leg, extending from the distal calf to the proximal thigh, with normal-appearing skin between lesions (Figure 1). Nodules were surrounded by hyperpigmented, slightly erythematous, violaceous skin and were cool-to-touch with evidence of prior drainage and ulceration in a few areas (Figure 2).

There was no inguinal lymphadenopathy or buboes. His vital signs were all within normal limits, without fever or tachycardia. Ultrasound of his leg was performed in the emergency department, given slight enlargement of his right leg compared to his left, and returned negative for deep vein thrombosis (DVT). He was admitted to the hospital, given concern for infectious etiology and monitoring of an associated acute kidney injury (AKI). On admission, his tacrolimus level was elevated at 13.9 ng/mL (goal 4-8 ng/mL), white blood cell count was 10.7 x 10^9^/L, and platelets were mildly low at 158 x 10^9^/L. His creatinine was elevated at 7.3 mg/dL from a baseline of approximately 2-3 mg/dL. His urinalysis was positive for glucosuria but negative for blood, RBC casts, protein, leukocytes, nitrite, or bacteria. Given the concern for an indolent infectious versus inflammatory process, an erythrocyte sedimentation rate (ESR) test was obtained and returned within normal limits. Additional labs were sent to evaluate for blastomycosis, histoplasmosis, hepatitis B and C, cryptococcus antigen, HIV, and TB via QuantiFERON gold, all of which returned negative. Since the patient was afebrile and did not exhibit any signs of systemic illness and recently completed a course of outpatient antibiotics, he was not started on empiric antibiotics while we awaited blood culture results, including AFB blood cultures, which remained no growth during his stay.

An urgent dermatology consult was placed, and the patient underwent a dermatologic biopsy on the first day of hospitalization. Histopathology results of the punch biopsy resulted as "dermal neutrophilic and granulomatous inflammation with focal necrosis, suggestive of infection. Grocott’s methanamine silver stain is negative for fungal organisms, and Fite and Ziehl-Neelsen stains are negative for mycobacteria." No bacterial or Nocardia colonies were isolated on culture, and no growth on fungal or AFB cultures. Given inconclusive biopsy results (not consistent with an autoimmune process but inconclusive for infection), general surgery was consulted, and the patient ultimately underwent incision and drainage (I&D) for further evaluation of these lesions. Pathology results returned as "skin necrosis and surface ulceration." Ultimately, tissue AFB cultures from the surgical I&D eventually returned with NTM. The patient was then started on imipenem, omadacycline, and clarithromycin and transitioned to omadacycline and clarithromycin on discharge at the direction of our infectious disease consult team. After discharge, cultures speciated as *Mycobacterium chelonae*. He continues to be monitored by Transplant Infectious Disease and has shown gradual resolution of the nodular rash on antimicrobial therapy. His serum creatinine also returned to baseline after admission, attributed to preceding trimethoprim-sulfamethoxazole therapy.

## Discussion

When approaching the immunocompromised patient with concern for skin and soft tissue infection (SSTI), it is important to maintain a wide differential. While Gram-positive bacteria staphylococci and streptococci remain the most common cause of SSTI, as with immunocompetent patients, the rates of atypical infection with Gram-negative bacteria, mycobacteria, and fungi are much higher in patients with suppressed immune systems [10]. As previously noted, SSTIs have been shown to have the highest prevalence of NTM extra-pulmonary infections [6]. Clinical clues indicating an atypical SSTI include nodules that are cool-to-touch and a lack of systemic illness. In these cases, atypical pathogens, including fungi, NTM, and non-pulmonary tuberculosis, should be considered. Special care should be taken to rule out necrotizing fasciitis, given its relatively high morbidity and mortality and coincident pulmonary mycobacterial infection. Empiric treatment of SSTI in the immunocompromised patient should be based on local prevalence factors. In general, empiric treatment should cover Gram-positive and Gram-negative bacteria, including pseudomonas, methicillin-resistant *Staphylococcus aureus* (MRSA), and fungi. Prompt incision, drainage, and tissue culture should be prioritized to narrow antimicrobial therapy as soon as possible. This is especially pertinent given the often prolonged time until cultures ultimately return positive for insidious pathogens, such as fungi and mycobacterium, which was the case with our patient.

Our patient ultimately grew *M. chelonae* on AFB culture taken from his surgical I&D sample. This was initially elusive despite the dermatology-obtained biopsy and culture earlier in his stay. This is despite the fact that *M. chelonae* is classified as a rapidly growing mycobacterium [11], as it is often difficult to grow and isolate on cultures. One study showed that *M. chelonae* was identified as the cause of 5.3% of NTM extra-pulmonary infections [6], while another identified only 6% of pulmonary NTM belonged to the species *Mycobacterium abscessus* or *M. chelonae* [12]. *M. chelonae* has also been shown to cause prolonged, subacute SSTIs in both immunocompetent and immunocompromised patients [13]. Therefore, NTM, including *M. chelonae*, should be considered in patients with SSTIs who have not responded to antibiotics, especially in patients who are immunocompromised, have had recent trauma-causing inoculation, or have undergone surgical intervention, given case reports of it causing surgical site infections and its ability to form biofilms on inadequately sterilized surgical equipment [14,15].

In our patient, we were also initially concerned about non-infectious etiologies such as vasculitis, given the associated AKI and non-blanching rash consistent with palpable purpura. However, given the lack of significant findings on his urinalysis, normal ESR, and unilateral presentation of the ascending rash/nodules, the clinical picture did not seem consistent with an autoimmune process. Therefore, we did not pursue workup with additional autoimmune labs. Ultimately, his AKI on chronic kidney disease was a red herring in his presentation, believed to be due to the trimethoprim-sulfamethoxazole use prior to admission and supratherapeutic tacrolimus levels.

Targeted antimicrobial therapy for NTM is ultimately based on speciation; however, many of the drugs used in these infections were not developed for this purpose; therefore, drug selection often requires infectious disease consultation and guidance [16]. A high index of suspicion for NTM infection is needed to appropriately diagnose and treat this type of infection.

## Conclusions

NTM infection can cause a broad range of pathology, especially in an immunocompromised patient. Therefore, when approaching this type of patient with concern for an SSTI, it is important to maintain a wide differential. Clinical clues that may indicate an atypical SSTI include nodules that are cool to touch, subacute in duration, and lack of systemic illness. In these cases, atypical pathogens, including fungi, NTM, and extra-pulmonary tuberculosis, should be considered. Obtaining tissue biopsy and histopathology may be of benefit if initial culture data are negative or inconclusive in order to avoid missing atypical pathogens, such as NTM. If NTM is identified as the source of infection, targeted antimicrobial therapy is ultimately based on NTM speciation.
